# Collecting clinical data in primary ciliary dyskinesia- challenges and opportunities

**DOI:** 10.12688/f1000research.9323.2

**Published:** 2016-10-03

**Authors:** Israel Amirav, Mary Roduta Roberts, Huda Mussaffi, Avigdor Mandelberg, Yehudah Roth, Revital Abitbul, Anthony Luder, Hannah Blau, Soliman Alkrinawi, Micha Aviram, Marta Ben-Ami, Moshe Rotschild, Lea Bentur, David Shoseyov, Malena Cohen-Cymberknoh, Eitan Kerem, Avraham Avital, Chaim Springer, Avigdor Hevroni, Husein Dabbah, Arnon Elizur, Elie Picard, Shmuel Goldberg, Joseph Rivlin, Galit Livnat, Moran Lavie, Nael Alias, Ruth Soferman, Heike Olbrich, Johanna Raidt, Julia Wallmeier, Claudius Werner, Niki T. Loges, Heymut Omran

**Affiliations:** 1 Department of Pediatrics, University of Alberta, Edmonton, Canada; 2 Department of Pediatrics, Ziv Medical Center, Faculty of Medicine, Bar Ilan University, Safed, Israel; 3 Department of Occupational Therapy, University of Alberta, Edmonton, Canada; 4 Schneider Children's Medical Center of Israel, Tel Aviv, Israel; 5 The Edith Wolfson Medical Center, Tel Aviv University, Holon, Israel; 6 Soroka Medical Center, Beer Shiva, Israel; 7 Rambam Medical Center, Haifa, Israel; 8 Hadassah-Hebrew University Medical Centers, Jerusalem, Israel; 9 Galilee Medical Center, Naharia, Bar Ilan Faculty of Medicine, Safed, Israel; 10 Assaf Harofeh Medical Center, Zerifin, Israel; 11 Shaare Zedek Medical Center, Jerusalem, Israel; 12 Carmel Medical Center, Haifa, Israel; 13 Sheba Medical Center , Tel Aviv University, Tel Aviv, Israel; 14 St Vincent Hospital, Nazaret, Israel; 15 Dana Children’s Hospital, Tel Aviv, Israel; 16 Department of General Pediatrics, University Children’s Hospital Muenster, Muenster, Germany

**Keywords:** response rate, cohorts, symptoms, questionnaire, clinical trial

## Abstract

**Rationale: **Primary ciliary dyskinesia (PCD) is under diagnosed and underestimated. Most clinical research has used some form of questionnaires to capture data but none has been critically evaluated particularly with respect to its end-user feasibility and utility.

**Objective: **To critically appraise a clinical data collection questionnaire for PCD used in a large national PCD consortium in order to apply conclusions in future PCD research.

**Methods:** We describe the development, validation and revision process of a clinical questionnaire for PCD and its evaluation during a national clinical PCD study with respect to data collection and analysis, initial completion rates and user feedback.

**Results:** 14 centers participating in the consortium successfully completed the revised version of the questionnaire for 173 patients with various completion rates for various items. While content and internal consistency analysis demonstrated validity, there were methodological deficiencies impacting completion rates and end-user utility. These deficiencies were addressed resulting in a more valid questionnaire.

**Conclusions:** Our experience may be useful for future clinical research in PCD. Based on the feedback collected on the questionnaire through analysis of completion rates, judgmental analysis of the content, and feedback from experts and end users, we suggest a practicable framework for development of similar tools for various future PCD research.

**At a Glance Commentary**:

**What is the key question?** It has been suggested that clinical data may be the first source of information when evaluating patients with PCD yet, only a few instruments to collect clinical data have been developed, and none have been critically evaluated.

**What is the bottom line?** Challenges in the development, validation and administration process of a clinical questionnaire for PCD are described.

**Why read on?** Based on the feasibility results, validity analysis and feedback collected, a newly revised validated questionnaire tool is now available providing opportunities for PCD research in additional patient populations and contexts.

**Abbreviations:** PCD- Primary Ciliary Dyskinesia, HVM- High Speed Video Microscopy, TEM-Transmission Electron microscopy, NO-Nitric oxide, IF-Immunofluorescence, LFT-Lung Function Tests, CT-Computed Tomography, RDS-Respiratory Distress Syndrome, CF-Cystic Fibrosis, ENT-Ear Nose & Throat.

## Introduction

Primary ciliary dyskinesia (PCD) is a genetic disease affecting the motile cilia in the respiratory system ^1– 3^. Clinical manifestations include neonatal respiratory distress, recurrent otitis media, sinusitis, and recurrent lung infections. As the clinical manifestations are variable and commonly encountered in children, the diagnosis is often delayed, particularly in the absence of situs inversus ^4, 5^. Clinical criteria have been suggested as an aid to PCD diagnosis ^5– 11^. Only a few large scale studies have used questionnaires to gather clinical information for PCD. For example, one of the largest pan-European studies ^5^ used a questionnaire to survey numbers and some characteristics of pediatric PCD patients (i.e., age, sex, age of diagnosis and presence of situs inversus) while the North American PCD consortium have used a much more detailed questionnaire in their prospective clinical studies ^8, 9^. A recent clinical internet-based tool is recently being developed ^10^.

As PCD clinical research is evolving, it is important that instruments for clinical data collection include relevant questions that generate accurate data, are valid for its intended purpose and use, are user friendly, and can be completed within a reasonable time.

Improved data collection instruments are of great importance, particularly for rare diseases such as PCD, where collaboration and data sharing is paramount. Unfortunately, none of the existing data collection tools have been critically evaluated. The purpose of this article is to describe the challenges of developing an improved clinical questionnaire for PCD, and evaluate its feasibility and potential utility for the end user. It is anticipated that this critical assessment of a PCD questionnaire will be useful for the development and refinement of similar tools to collect clinical data in various PCD studies.

## Methods

### Context for questionnaire development – NIPC study

In 2011 we decided to characterize the clinical features of PCD patients in Israel. A prospective National Israeli PCD Consortium (NIPC) study was conducted between the years 2011–2013 in subjects presenting with the *typical* clinical phenotype of PCD in 14 pediatric pulmonology centers (for details see ref. 12). Ethical approval was obtained from Institutional Review Boards at each centre that collected patients’ data (Ziv Medical Center, Schneider Children’s Medical Center, Edith Wolfson Medical Center, Soroka Medical Center, Hadassah-Hebrew University Medical Centers, Rambam Medical Center, Western Galilee Hospital, Saint Vincent De-Paul Hospital, Assaf Harofeh Medical Center, Shaare Zedek Medical Center, Edmond & Lili Safra Children’s Hospital, Sheba Medical Center, Carmel Medical Center, Dana Children’s Hospital) as well as from the Israeli Ministry of Health. and all patients and guardians signed written informed consent. The study was registered on ClinicalTrials.gov (NCT 01070914). To complement the clinical phenotypic data and in order to verify the diagnosis, the subjects had a comprehensive study visit whereby they also underwent a series of tests including nasal NO (nNO), nasal brushing of samples for transmission electron microscopy (TEM), immunofluorescence (IF), high-speed video microscopy (HVM) and blood sampling for genetics. In this study, the presence of at least 2 abnormal results for these tests were used as criteria to define PCD ^3^. To capture the most accurate clinical information in this study, a special questionnaire had to be developed.

### Development, validation and revisions of the questionnaire

Only a few questionnaires have been previously used to specifically collect clinical data in PCD and none of them as far as we know, have been systematically and critically evaluated ^5– 11^.

To validate the content of the NIPC questionnaire, a national expert panel was created. It consisted of three pediatric pulmonologists, one adult pulmonologist and one ENT surgeon with expertise in PCD. All members had been in practice at least 15 years after certification and have run PCD clinics for at least 10 years.

The initial draft of the NIPC questionnaire was developed by the panel members after reviewing existing questionnaires ^5, 6, 8– 11^ and selecting content. Across multiple iterations the panel provided feedback regarding the content relevance and representativeness with respect to the clinical presentation of PCD. Discussions between panel members were conducted electronically and by conference calls. Based on feedback of the panel, modifications to the questionnaire were made after each iteration. The major concern of the panel was the excessive length and detail of the initial draft. It was felt that both organizational and content changes had to be addressed in order to make completion of the questionnaire feasible within a reasonable amount of time.

As the proposed study had planned to enroll subjects who were clinically suspected of having PCD, there were many items the panel found unnecessary or irrelevant. For example, history probing about meconium aspiration was considered irrelevant for the diagnosis of PCD. Likewise, items regarding disorders of family members extending to relatively remotely-related members (e.g., biological maternal grandfather) were also deemed unnecessary. While genetic predisposition is important in PCD, the panel felt that there are more direct ways to obtain such information (e.g. questions on consanguinity and pedigree creation). Another problem identified by the panel was overlapping content. Many questions were repeated in various sections while providing similar information. For example, radiological evidence for sinus abnormality appeared both in the medical history and in the test sections. Consensus on questionnaire content was reached after approximately 10 iterations. During the revision process, we tried to balance the need of adding new clinical data with reducing the burden of a long and impractical questionnaire.

The main sections of the revised NIPC questionnaire were: A. Demographic details (9 items), B. Family history (8 items), C. Past medical history (52 items) and D. Physical examination and basic tests (radiology, spirometry, sputum etc.) (38 items). Response options were in the form of: YES/NO/Do not know; commentary or multiple choice. Some items included subsidiary questions. For example, when enquiring about the presence of chronic cough, a subsidiary question followed asking when the symptom began.

Beyond the history regarding PCD diagnosis, the questionnaire included several "rule out" questions such as normal sweat tests for cystic fibrosis.

### Questionnaire completion, distribution and collection

The NICP questionnaire was emailed to the local principal investigator (PI) of each center (n=14) approximately one week prior to a scheduled visit. The local PI was asked to forward the questionnaire to the other physicians in their group (e.g. a center may have three physicians where each one physician is responsible for the care of a few patients with PCD). The physicians were asked to print out the questionnaire and complete it for each of their scheduled patients within one week.

The national PI and 2 or 3 research assistants (RA) joined the local PIs during actual patient visits (study sessions) in order to assist in the ancillary diagnostic test procedures (nasal brushing, video-microscopy etc.). There were 20 study sessions in all of the participating centers spanning an almost two-year period during which some centers had more than one session. Completed questionnaires were physically collected from each center by the study PI (IA) at the end of each session. Questionnaires were scanned and reviewed for missing or unclear data by the study PI and a RA. Unanswered questions were noted and a reminder to complete the questions was e-mailed to the local PI within 2 weeks of collection.

A second attempt to complete unanswered questions was done by transferring the received questionnaire data to an Microsoft Excel 2010 table, marking all missing or unclear answers, and e-mailing the table to the responsible physician. When a third e-mail reminder was necessary, the first author also personally phoned the local PI at each center to encourage them to complete the unanswered questions.

### Data analysis of completed questionnaires to evaluate feasibility and utility

***Quantitative analysis.*** Completion rates of each item on the questionnaire, as an indicator of feasibility, were calculated as a percentage, and then averaged across items for each section of the questionnaire (Microsoft Excel 2010). Completed items were coded as 1, whereas uncompleted items were coded as either relevant (code 2) or not relevant to the particular patient (code 3, e.g. an uncompleted item about fertility in a child). If an uncompleted item included a N/A answer, it was also coded as 3. Data were analyzed for the total group, and for those eventually diagnosed with PCD.

***Qualitative analysis.*** Qualitative evaluation of the questionnaire feasibility and utility was completed from two post–hoc perspectives. The first was based upon guidelines on best practices for questionnaire development ^13^. These guidelines focus on: a) features of the questionnaire (visual presentation, language and format, mode of administration), b) characteristics of the participant completing the questionnaire (their workflow, their degree of cooperation, their relationship with the researcher), and c) interaction of the participant with the questionnaire (i.e., what must the participant do to and data sources required to complete the questionnaire?).

The second was the physicians’ ***post-hoc user feedback***. This feedback was collected through electronic communications, oral discussions and through an electronic survey using http://www.questionpro.com/a/listSurveys.do.

The survey is included in under Data availability ( Dataset 1). Participants were first presented with specific items with a low completion rate and then were asked to suggest potential reasons for this. They were also surveyed about more general topics such as relevancy and representativeness of the items for PCD, preference for electronic vs paper records, time required to complete the questionnaire and future use in their practice.

PCD- Post Study Feedback questionnaire to physicians Click here for additional data file. Copyright: © 2016 Amirav I et al. 2016 Data associated with the article are available under the terms of the Creative Commons Zero "No rights reserved" data waiver (CC0 1.0 Public domain dedication).

### Validation analysis

Content validation was conducted as previously described during the development stage. To assess internal consistency, we first computed frequencies and descriptive statistics for all items using IBM SPSS Statistics for Windows, Version 23.0 ^38^ Attention was paid to the proportion of responses coded as not completed or unknown and not relevant. Items with a large proportion of responses coded as not relevant were often subsidiary questions to a lead question. Judgments were made regarding the added value of retaining these subsidiary questions for the analysis of internal consistency, over and above the information already provided by the lead question. Items with a large proportion (i.e., greater than 0.25) of responses coded as not completed or unknown also warranted a closer look at the item with judgments made on whether it should be included in further investigations of internal consistency. Internal consistency for the two major subsections (medical history and physical examination) was then examined using Cronbach’s alpha. Missing data were handled using the default option of listwise deletion in IBM SPSS Statistics for Windows, Version 23.0 ^38^.

Taken together, the results were used to inform revisions to the PCD questionnaire.

## Results

### Quantitative analysis (Completion rates)

Twenty-two physicians in 14 centers completed questionnaires for 173 subjects (of which 104 were eventually confirmed as PCD). Table 1 summarizes the initial completion rates for each subsection of the PCD questionnaire by group (i.e., total, PCD, non-PCD).

**Table 1.** Initial Completion rate of various questionnaire sections. Item codes: 1=completed, 2=not completed yet item relevant, 3=not completed and item not relevant.

		Total (n=173)					PCD (n=104)					non-PCD (n=69)			
	Section	A	B	C	D		A	B	C	D		A	B	C	D
**Item** **Code**															
**1**		96	78	56	57		97	80	59	59		95	75	52	55
**2**		4	10	13	11		3	9	13	10		5	10	13	11
**3**		0	13	31	32		0	11	29	31		0	15	35	34

**Figure 1.**
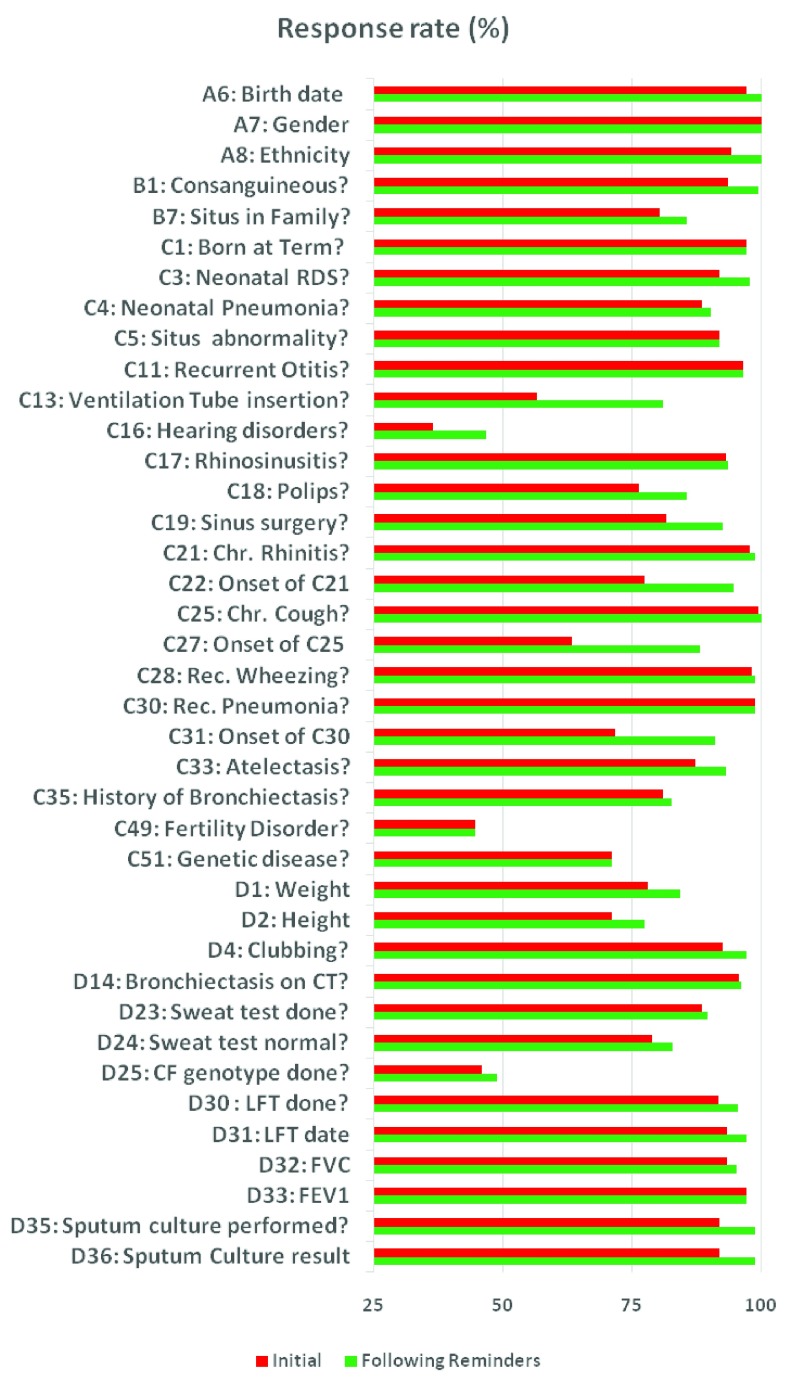
Changes in response rate (%) between initial completion (red columns) and following reminders (green columns) for selected questions. All questions ending with a (?) had yes/no/unknown answers. The rest had free text answers (e.g. birth date). A, B, C and D are the questionnaire sections. Abbreviations: NRDS-Neonatal Respiratory Distress Syndrome; LFT- Lung Function Test; FVC- Forced Vital Capacity; FEV1-Forced Expiratory Volume at 1 ^st^ second; CT- Computed Tomography; CF- Cystic Fibrosis.

Overall, the average initial response rate was 82% and increased to 88% following two reminders. Subsections A (range=95–97%) and B (range=75–80%) had high initial completion rates. Subsections C (range=52–59%) and D (range=55–59%) had lower completion rates (p<0.01). This pattern of completion rates corresponded to items that required recall of readily available information about the individual and their past/family medical history (subsections A and B), whereas subsections C and D required more specific tests information. No significant differences in response rates were observed between PCD and non-PCD groups. Figure 1 illustrates the changes in response rate (%) between initial completion and following reminders for major individual questions.

Among the uncompleted items, seven were identified where a response was relevant and required (i.e., item coded as 2). One item was in the family history subsection B, five items were in the medical history subsection C, and one item was in the physical examination subsection D. Two of these items, both in the medical history subsection C, had a non-response of > 50%. The first of these items (64% non-response rate) enquired about the patient’s hearing loss with sub-questions to identify its nature: conductive, neuronal, or unknown. A potential reason why this item might not have been answered is that the response options were not exhaustive. If the patient did not have hearing loss, then none of the response options would have been the appropriate answer. Therefore, leaving the item unanswered was one course of action. The second item with >50% non-response was a sub-question about lung transplant which was found to be confusing. It was only post-hoc analysis that revealed the problematic nature of these items.

Five uncompleted yet relevant items had non-response rates between 25–50%. For example, one item enquired whether the patient had family members who suffered from any of 12 various symptoms. Although the symptoms were specific (e.g., chronic rhinitis, fertility disorders), patients may not have known their family history to that degree of detail or the information may not have been entered in the chart. Other questions with low response rate asked for the results of specific tests such as the sweat chloride concentration. If this information was not readily available from the chart or from the patient, this question would not have been answered.

## Qualitative analysis

### Evaluation based on best practice guidelines for questionnaire development

***Features of the questionnaire.*** There were two areas which could have been improved upon. First was consideration of terminology used and its consistency in interpretation across participants. For example, questions about “hearing loss”, and its sub-categories may not have a universally accepted definition thus resulting in differing interpretations ^11^. Second was consideration of questionnaire length. At 98 questions, the NIPC questionnaire was an improvement in length when compared with previously used questionnaires increasing the chances of collecting complete data ^14^.

***Characteristics of participants.*** In terms of the participant group, the questionnaire content and length may have impacted completion given how busy physicians’ practices are. However, continuous email and phone support provided by the study PI to the physicians likely helped to achieve completion rates of almost 90% using personal reminders.

***Interaction between the participant and questionnaire.*** The effort to complete the clinical questionnaire ranged from minimal (e.g., completing demographic section) to at least moderate (e.g., completing family history or physical examination). Answers for some questions required specific data for which information within the records/charts were not easily available. In these cases, the physician had to complete the questionnaire during or after the actual patient visit. Analysis of free text answers was challenging and was not contributory in most cases.

### Evaluation based on feedback from end users

Seventeen out of the 22 end users (i.e., physicians) provided feedback and identified reasons for low completion rates of some items. For example, 70% identified lack of available data as a reason for a low completion rate of the question about polysplenia. Twelve percent suggested that this item was irrelevant. A low completion rate for the item enquiring about *onset* of chronic cough and *onset* of wheezing were attributed to lack of available data in 53% of responders. This was also the case with “hearing disorders” (response rate of 47%). Fifty-eight percent thought that the fertility question was irrelevant to their subjects (children). Most (63%) reported that it took them less than an hour to complete the questionnaire in one session; 31% reported they required several sessions to do it; 81% preferred the use of electronic rather than paper versions and 75% said they will use the questionnaire as a diagnostic tool in their practice.

### Internal consistency

Several items had a high proportion (>50%) of responses coded as not relevant. All these items were subsidiary questions to lead questions and were carefully reviewed before internal structure analysis. For example, the lead question C5 asks whether situs inversus is present. The follow-up questions C6 to C9 had 67.1% responses coded as not relevant, likely because situs inversus was not present in their patient. The PI reviewed these items individually and determined that question C5 captures the presence of this primary feature (i.e., situs inversus) within the patient sample whereas questions C6–C9 are subsumed conceptually by C5 and could be excluded from further analysis of internal structure. For the medical history subsection, Cronbach’s alpha was 0.63 and for the physical examination/tests subsection Cronbach’s alpha was 0.72.

## Discussion

Previous studies have collected some clinical data in describing their PCD population without the use of a specific questionnaire. To better contextualize our study within the current state of acquiring clinical data in PCD, we systematically reviewed studies published in the English literature over the past 5 years (2010–2015) that have detailed clinical characteristics of PCD patients. In brief, two authors (IA, AM) independently searched MEDLINE and EMBASE followed by additional bibliographies of all selected studies. Disease-specific terms (primary ciliary dyskinesia, Kartagener syndrome, immotile ciliary syndrome, immotile cilia syndrome) were combined with clinical data-specific terms (clinical features, characteristics, symptoms, gender, age, family history, consanguinity, ethnicity, pneumonia, rhinitis, otitis, situs, bronchiectasis, atelectasis, clubbing, polyps, sinusitis) as search parameters.; Inclusion criteria included all studies published before the start of this search on December 10, 2015 on PCD patients (n≥30) that systematically detailed clinical characteristics. Categories of clinical items collected in each study were then tabulated ( Table 2).

**Table 2.** Clinical items collected in PCD studies.

1St Author (year, Ref.)	Item/ Major outcome/purpose	No. of patients	Gender	Ethnicity	Consanguinity	NRDS	Bronchiectasis	Situs (laterality defects)	Otitis	Ventilation t./ hearing loss	Rhinitis (all types)	Cough* (all types- chronic, recurrent, wet..)	Pneumonia/LRTIs	Sinusitis	Family history of Resp problems/PCD	Nasal Poliposis	Asthma Wheeze	Clubbing	Atelectasis
**Davis S (2015**, ^**15**^)	Phenotype- Genotype correlations	118	+	+			+	+	+		+	+	+						
**Djakow J (2015, ^16^)**	Phenotype- Genotype correlations	38	+			+		+	+		+	+	+	+					
**Hosie P (2015, ^17^)**	General clinical	84				+	+	+	+		+	+	+	+	+			+	
**Yiallouros P (2015, ^18^)**	General clinical	30	+			+	+	+	+	+	+	+	+			+		+	
**Cao Y (2015, ^20^)**	General clinical	134	+	+	+	+	+	+	+	+	+	+	+	+	+	+			+
**Boon M (2014, ^19^)**	TEM Description/correlations	168	+	+	+	+	+	+	+	+	+	+	+	+	+	+	+	+	
**Cohen-Cymberknoh M** **(2014, ^21^)**	Comparison with CF	34	+			+	+	+	+					+					
**Mulloowney T (2014, ^22^)**	Neonatal characteristics	46	+			+	+	+											
**Vallet C (2013, ^23^)**	TEM Description/correlations	60	+		+	+	+	+	+		+			+	+	+			
**Kim R (2013, ^24^)**	Phenotype- Genotype correlations	52	+	+	+	+	+	+	+					+					
**Busquets R (2013, ^25^)**	TEM Description/correlations	34	+			+	+	+					+				+		+
**Demarco RC (2013, ^26^)**	TEM Description/correlations	35	+				+	+	+		+								
**Zietkiewicz E (2012, ^27^)**	Phenotype- Genotype correlations	213					+	+	+				+	+					
**Blanchon S (2012, ^28^)**	Phenotype- Genotype correlations	43	+	+	+	+	+	+	+			+	+			+			
**Knowles M (2012, ^29^)**	Phenotype- Genotype correlations	195	+	+	+	+	+	+	+					+					
**Piferri M (2011, ^30^)**	Sinus Radiology	41	+			+		+							+					
**Sommer J (2011, ^11^)**	ENT manifestations	44	+					+	+	+	+			+		+			
**Theegarten D (2011, ^31^)**	TEM Description/correlations	125	+					+	+				+						
**Noll EM (2011, ^6^)**	General clinical	46	+			+	+	+	+		+		+	+			+		
**Marthin JK (2010, ^32^)**	LFT	74	+			+	+	+	+		+	+		+	+				
**Zietkiewicz E (2010, ^33^)**	Phenotype- Genotype correlations	185					+	+	+		+		+	+					
**Prulière-Escabasse V** **(2010, ^34^)**	ENT manifestations	58					+	+	+	+									
																			
	Total (n)	**1799**																	
	Item collected in # of papers		**18**	**6**	**6**	**14**	**19**	**22**	**19**	**5**	**12**	**8**	**12**	**14**	**5**	**6**	**3**	**3**	**2**
	Item collected in % of papers		**82**	**27**	**27**	**64**	**86**	**100**	**86**	**23**	**55**	**36**	**55**	**64**	**23**	**27**	**14**	**14**	**9**

Abbreviations: NRDS-Neonatal Respiratory Distress Syndrome LRTI-Lower respiratory Tract Infection LFT- Lung Function Test TEM-Transmission Electron Microscopy ENT-Ear, Nose & Throat CF- Cystic Fibrosis

Differences in judgment were resolved by consensus. Twenty-two studies ^6, 8, 11, 15– 34^ totaling 1799 patients with PCD met our inclusion criteria. The most striking finding of our review was the wide variability and lack of standardization in collecting clinical data in PCD studies. While there were a few clinical items that were collected frequently [e.g., situs (in 100% of studies), age (91%), otitis and bronchiectasis (in 86%)], there was no consistency with many other clinical items. This review was very informative and supported motivation for our work in developing improved instruments (such as a questionnaire) for collecting clinical data in PCD. Here we present the first report and discuss what we have learned about the development and field evaluation of a clinical questionnaire for PCD. As clinical data collection is fundamental not only for clinical care but also for registry development and studies such as cohort, interventions, genetic and epidemiological, these lessons are applicable across many other settings.

Questionnaires must have a high response rate since the ultimate goal is to obtain accurate and high quality clinical data ^14^. The quantitative analysis in the present study identified questions that had low response rates or might have been completed inconsistently. Reasons for low completion included issues such as perceived relevance of the item to the patient, problematic wording and formatting of the question, information not readily available in the patient chart, and difficulties of the patient or parents recalling pertinent medical information, the latter already identified in a previous PCD study ^35^. Questions with low response rates could be given back to the end users or expert panel to render a judgment on question relevance or format.

Based on guidelines for questionnaire development, we suggest that like any other diseases, PCD questionnaires should be developed with target users in mind, considering their characteristics and workflow to assure as high a response rate as possible while minimizing the burden of participation. Despite the “generic” nature of the clinical questions, the specific purpose of the clinical questionnaire and instructions for its completion should still be explicit and clear in any future individual study. Given that some questions will require very specific information, access to medical charts should be encouraged upfront. Since the present study targeted subjects with a high suspicion of PCD, the expert panel suggested that in such populations questions be more focused toward specific PCD symptoms taking into account that these subjects have probably undergone considerable previous evaluation and, in particular, queries regarding associations to respiratory illness (e.g., removing previous probing about pancreatic insufficiency).

Before distribution to end users, the tool should be pilot tested at least once with a small sample representative (e.g., clinicians or research assistants) ^14^. Pilot testing should be done to check on formatting, clarity of wording, content coverage and to test if the questions are working in the ways originally intended. One group of testers should typically be experts in the field to inform content validity whereas another group should be the target end users. The more feedback received about the questions from these two groups, the more likely the tool will be in a form that is relevant to the data collection purpose and readily acceptable to participants in order to achieve as high as possible completion rates.

The end-user feedback in this study helped us to revise many questions. Moreover, based on this feedback, analysis of items coded as 2 (i.e., not completed but relevant) suggested that while content validation was adequate, the expert panel did not sufficiently attend to other methodological aspects such as item wording, clarity, interpretations and structure. These issues might have been better addressed by questionnaire experts. Of interest, but not surprising, is the fact that most of the end users preferred electronic rather than paper versions. Such an internet based PCD registry, likely to reduce cost and time ^36^ is indeed being recently developed ^10^.

Over time, ongoing analysis of question completion rates in various PCD populations and contexts may show that the items flagged in this paper continue to provide low response rates. This empirical evidence suggests further discussion as to whether these items should remain in the questionnaire or at the very least, discussion on how to improve response rates beyond what was outlined previously.

Although beyond the scope of the present study, it would be useful in the future to correlate the various questions with individual patients’ data and to evaluate their predictive diagnostic or prognostic value. For "real life" applications, the questionnaire can be modified and adapted. The items can be streamlined to represent the specific goal in mind. Recent papers have identified the role of few clinical features that are highly associated with proven PCD ^7, 9^. Using these suggested more selective items and removing all others from our proposed questionnaire, may indeed can substantially improve the response rate as well as the completion time.

Similarly, for intervention studies there is clearly no need to include many of the Demographics (section A), Family history (section B) and Medical history (section C) items, but to focus on specific modifiable items (for example pulmonary function tests).

## Conclusion

This is the first critical appraisal of a clinical questionnaire for PCD. Based on analysis of questionnaire completion rates, validation of the questionnaire, literature review and feedback from both experts and end users, we have now developed a shorter, clearer and more user-friendly updated generic version. This newly revised version may be freely downloaded from the Data availability section of this paper ( Dataset 2) by any researcher and/or clinician interested in collecting clinical data about PCD. Indeed, it was recently used in a multi-national PCD European study ^37^. Clinical research in PCD will gain much benefit from future use and further validation of this tool with additional patient populations and contexts.

Newly (2016) Revised Suggested PCD Questionnaire Click here for additional data file. Copyright: © 2016 Amirav I et al. 2016 Data associated with the article are available under the terms of the Creative Commons Zero "No rights reserved" data waiver (CC0 1.0 Public domain dedication).

## Data availability

The data referenced by this article are under copyright with the following copyright statement: Copyright: © 2016 Amirav I et al.

Data associated with the article are available under the terms of the Creative Commons Zero "No rights reserved" data waiver (CC0 1.0 Public domain dedication). 

*F1000Research*: Dataset 1. PCD- Post Study Feedback questionnaire to physicians, 10.5256/f1000research.9323.d131910 ^39^

*F1000Research*: Dataset 2. Newly (2016) Revised Suggested PCD Questionnaire, 10.5256/f1000research.9323.d131911 ^40^
