# Supplementary material for: Collecting clinical data in primary ciliary dyskinesia- challenges and opportunities
Source: F1000Res. 2016 Oct 3;5:2031. Originally published 2016 Aug 18. [Version 2] doi: 10.12688/f1000research.9323.2 (PMC5022703; doi:10.12688/f1000research.9323.2)
Supplement: Peer review history

# Peer review: Referee response for version 2

**DOI:** 10.5256/f1000research.10392.r16808

**Published:** 2016-10-05

**Authors:** Bruce Rubin

**Affiliations:** 1 Department of Pediatrics, Virginia Commonwealth University School of Medicine, Richmond, VA, USA

I have read this submission. I believe that I have an appropriate level of expertise to confirm that it is of an acceptable scientific standard.

# Peer review: Referee response for version 1

**DOI:** 10.5256/f1000research.10039.r16025

**Published:** 2016-09-09

**Authors:** Zorica Živković

**Affiliations:** 1 Faculty of Pharmacy Novi Sad, Business Academy Novi Sad, Novi Sad, Serbia

I have read carefully the article on collecting clinical data in primary ciliary dyskinesia, the usefulness and comprehensiveness of the questionnaire used. I admire great effort and workload of the authors to set up this questionnaire and to approve its applicability in real life. From that point the topic has achieved its main goal. The other point is the low feedback responses and related questions that have been analyzed. Very interesting and valuable issues were raised. All of us working with questionnaires spent most of our time trying to avoid low response rate. Since this paper has no input on clinical diagnosis or treatment option in primary ciliary dyskinesia I would suggest this key problem to be pointed out. What would be the possible opportunities (as noted in the title) for solving this problem?

 In the methodology section, I would like to add the way that physicians filled up the questionnaires with their patients, precise time frame that they have been used including the time for explanations and clearing up the complicated or hardly understandable questions. Having in mind very detailed time consuming questionnaire, the importance should be in national based registry making, which should be pointed as well.

 I must say that reviewing this manuscript was real honor and I strongly support its publishing.

I have read this submission. I believe that I have an appropriate level of expertise to confirm that it is of an acceptable scientific standard.

# Response

**Published:** 2016-09-16

**Authors:** Israel Amirav

Thanks for your favourable comments. The point of low response rate is well appreciated. We believe this is a major concern in all questionnaire type studies. We have highlighted its importance as we made it one of the major outcomes of our study (see quantitative analysis).

 The comments about opportunities for clinical diagnosis and possible treatments are correct yet these were beyond the purpose of this study. We nevertheless, just before the conclusion section we mentioned that "Although beyond the scope of the present study, it would be useful in the future to correlate the various questions with individual patients’ data and to evaluate their predictive diagnostic or prognostic value."

 With regard to the time required to fill the questionnaire, we have partially referred to it in our evaluation based on feedback from end users: "Most (63%) reported that it took them less than an hour to complete the questionnaire in one session; 31% reported they required several sessions to do it;"

 The reviewer is correct that we have not specifically asked about the time needed for explanation and clearing up complicated questions. A good point for evaluation of future instruments!

 We completely agree that the questionnaire could be also used in national registries and we have mentioned it in the 2nd paragraph of the discussion: "As clinical data collection is fundamental not only for clinical care but also for registry development and studies such as cohort, interventions, genetic and epidemiological, these lessons are applicable across many other settings."

# Peer review: Referee response for version 1

**DOI:** 10.5256/f1000research.10039.r15746

**Published:** 2016-08-19

**Authors:** Bruce Rubin

**Affiliations:** 1 Department of Pediatrics, Virginia Commonwealth University School of Medicine, Richmond, VA, USA

This is an outstanding study demonstrating validation of a PCD clinical questionnaire by an expert group of clinicians from Israel. The data are well presented and the results will be valuable to clinicians caring for children and adults with PCD.

 However this is still quite a long questionnaire (98 questions and an hour to complete) and the authors have not made it clear how this questionnaire is to be used:

1. As a diagnostic screening tool this could be stratified and streamlined to identify criteria sufficient for patients to be referred for additional testing such as nNO, TEM and video, or genetics.
2. As a tool to monitor disease progression, many of the background questions would be redundant on repetition.
3. As a tool to evaluate the outcomes of specific interventions (results of therapy) many of these questions are irrelevant.
4. As a "general PCD questionnaire" it is long and time consuming to complete and may have limited application for an individual patient or clinician.

I would appreciate it if Prof Amirav would discuss how he would see this questionnaire used in "real life" and if shortened versions might be more relevant for specific clinical applications.

I have read this submission. I believe that I have an appropriate level of expertise to confirm that it is of an acceptable scientific standard.

# Response

**Published:** 2016-09-16

**Authors:** Israel Amirav

We thank Prof Rubin for his thoughtful comments. We completely agree that for "real life" applications the questionnaire can be modified and adapted. In any of these situations, the items can be streamlined to represent the specific goal in mind. Recent papers have identified the role of few clinical features that are highly associated with proven PCD (7,9). Using these suggested more selective items and removing all others from our proposed questionnaire, may indeed can substantially improve the response rate as well as the completion time.

 Similarly, for intervention studies there is clearly no need to include many of the Demographics (section A), Family history (section B) and Medical history (section C) items, but to focus on specific modifiable items (for example pulmonary function tests).

 We have now included this notion in our revised discussion.

# Response

**Published:** 2016-10-04

**Authors:** Bruce Rubin

**Affiliations:** Department of Pediatrics, Virginia Commonwealth University School of Medicine, USA

Israel - Are you comfortable proposing what these modified questionnaires might consist of? Have you enough data from this validation study to do this? It would be very useful.

 Thanks

 Bruce
